# Acceptance of sugar reduction in yoghurt among Moroccan population

**DOI:** 10.11604/pamj.2017.28.310.12257

**Published:** 2017-12-15

**Authors:** Hasnae Benkirane, Youness Taboz, Nada Benajiba, Yasmine Guennoun, Abderrazzak Khadmaoui, Amina Bouziani, Habiba Bajit, Khalid El Kari, Nawal Bentahila, Amina Barkat, Hassan Aguenaou

**Affiliations:** 1 Unité Mixte de Recherche en Nutrition et Alimentation URAC 39 (Université Ibn Tofaïl–CNESTEN), RDC-Nutrition, Kénitra, Maroc; 2 Clinical Nutrition Program, Department of Health Sciences, College of Health and Rehabilitation Sciences, Princess Nourah Bint Abdulrahman University, Riyadh, KSA; 3 Laboratoire de Génétique et Biométrie, Faculté des Sciences, Kénitra, Maroc; 4 GANIM, Groupe de Recherche en Nutrition Infantile, Maroc; 5 Equipe de Recherche en Santé et Nutrition du Couple Mère-Enfant, Faculté de Médecine et de Pharmacie de Rabat, Université Mohammed V de Rabat, Maroc

**Keywords:** Sugar reduction, acceptance, yoghurt, Morocco

## Abstract

**Introduction:**

Morocco has recently developed a plan of reducing sugar consumption to reinforce prevention of non-communicable diseases and to contribute to the achievement of global voluntary targets for non-communicable diseases set by ICN2 by 2025. The objective of the present study was to assess acceptance of yogurts with different percentage reduction of sugar by the Moroccan population.

**Methods:**

A total of 201 participants (age > 15 y.) were recruited to determine the level of sugar reduction in yogurt. Sucrose was added to a plain yoghurt in the following different concentrations 166.5; 149.8; 133.2; 116.5; 99; 83.2 mM/l, corresponding to the reduction of sugar of 0%, -10%, -20%, -30%, -40% and -50%, respectively, compared available yogurt in local market. Overall, the acceptability scores of the different yoghurts were based on liking, “Just About Right” (JAR) and purchase intent scales was used to score the different yoghurts.

**Results:**

Yogurts containing -20% and -30% added sugar were highly accepted by 81% and 74% of respondents. Based on JAR score, yoghurt with 20% (133.2mM/l) and 30% (116.5 mM/l) reduction were considered as “Just about right” by 42.7% and 44.3% respectively. Best average score of purchase intent was obtained for sucrose concentration of 149.8 mM/l. 35.8% and 40.3% for yoghurt with sucrose concentration of 133.2 mM/l and 116.5 mM/l respectively.

**Conclusion:**

The finding from this study indicated that yogurts containing -20% and -30% added sugar were most accepted by respondents. Advocacy before dairy industry to have them commit towards sugar reduction in yogurt is needed, in order to help achieving the national sugar reduction strategy in Morocco.

## Introduction

Morocco has recently developed a plan of reducing sugar consumption in order to reinforce prevention of non-communicable diseases. This action has been taken to contribute to the achievement of global voluntary targets for non-communicable diseases set by the Second International Conference on Nutrition ICN2 by 2025 [1]. In fact, excessive sugar consumption from food is a real worldwide concern as it is currently well established that it leads to various negative effects on health such as obesity, insulin resistance, hypertension and Type 2 diabetes. Thus, according to new World Health Organization (WHO) guidelines published in 2015, it is recommended to limit the intake of free sugars to less than 10% of total energy intake in both adults and children [2]. WHO suggests a further reduction of the intake of free sugars to below 5% of total energy intake, or about 25 grams (6 teaspoons) per day. This recommendation is intended not only to the consumer but also to the food industry, which should also consider sugar content reduction in processed foods [3]. However, such a change in food processing poses a major challenge as products need to be reformulated with less sugar while maintaining acceptance and attractiveness by consumers. With this regard, various studies reported that consumers, including children, adults as well as the elderly, preferred food with high content in sucrose [4-6]. Even though, dietary preferences vary according to age and type of food, sugar is always needed by human body to produce energy and maintain metabolism [3, 5, 7]. Yoghurt is perceived by consumers as a healthy food due to its high content in calcium and presence of active microorganisms [8]. Therefore, this positive perception shouldn't be affected by high amount of sugar or use of artificial ingredients such as sweeteners. Although, sugar in yoghurt is essential for improving taste, texture, body, viscosity and moisture retention [8]. Gille et al (2012) demonstrated that 51% of Swiss respondents stated that available flavored yogurts in the market were very sweet [9]. Yogurt consumption in Morocco has remarkably increased. It was 3.4 and 9.2 kg/p/year among medium and low socio-economic class respectively in 2003. In 2015, it reached 5.7 and 17.2 kg/p/year among these two populations [10]. Since yogurt preferences are mainly influenced by texture, flavor and taste, then the concentration levels of sugar in yogurts is crucial [11-14]. Thus, consumer satisfaction and acceptance of yogurt may hinder its beneficial effect on health [11, 12]. Therefore, the objective of the present study was to assess acceptance of yogurts with different percentage reduction of sugar among Moroccan population using taste, “Just About Right” (JAR) and purchase scales, being the currently available yogurt in local market as a reference.

## Methods

**Subjects**: A total of 201 participants (age > 15 years) were recruited to determine the level of sugar reduction in yogurt. Males constituted 48.3% and females 51.7%. Information on age (years), gender and frequency of consumption of yogurt were collected using a valid questionnaire [12]. Individuals who consumed less than one cup of yogurt per month or had preference to plain yogurt were excluded from the study.

**Sample preparation**: Plain yoghurt (0 mM sucrose/l; 6.4g carbohydrates/100g yoghurt) was chosen as reference for 0% added sucrose, while the sweet yogurt (166.5 mM sucrose/l; 12.1 g carbohydrates/100 g yoghurt) was chosen as the reference of 100% added sugar, as this is the usual concentration of sugar in yogurt available in Moroccan local market. Sucrose was added to a plain yoghurt in the following different concentrations 166.5; 149.8; 133.2; 116.5; 99; 83.2 mM/l, which corresponded to the reduction of sugar to obtain different sweetness levels corresponding to a reduction of 0%, -10%, -20%, -30%, -40% and -50%, respectively. The mixtures were homogenized and then cooled at 5°C for 5 to 7 days until descriptive sensory analysis were conducted [12].

**Sensory analysis**: Participants were requested to taste the yogurts and evaluated their overall liking based on taste scale. It consisted a 9-points hedonic scale (1 = I extreme dislike to 9 = I extreme like). The 9 points were grouped into 3 categories: 1 = I dislike, 2 = I either like nor dislike and 3 = I like. Sweetness intensity was assessed using the JAR (Just About Right) 9-points scale (1-4 = not sweet enough, 5 = just about right, 6-9 = too much sweet). Purchase intent was scored on a 5-point scale (1 = would definitively not buy, 5 = would definitively buy). Weekly frequency of yogurt consumption was also determined. Each participant evaluated 7 yogurt samples, which were presented in 3-digits coded [15]. Three samples were evaluated per cold session and in a random order. The order of presentation was reversed between the test sessions using the 3AFC blind method [16-18]. Rinsing between samples was done by flat water [12].

**Statistical analysis**: Statistical analysis was performed using SPSS (version 21). The data were analyzed using ANOVA, at a p-value ≤0.05, mean values were further compared using FISHER test. Scores distribution frequency was calculated for the 3 used scales (taste scale, JAR scale and purchase intent scale). The percentage (%) of scores in each category was calculated and Freidman's Chi^2^ test was applied to assess the significance between the different categories.

## Results

**Figure 1:**
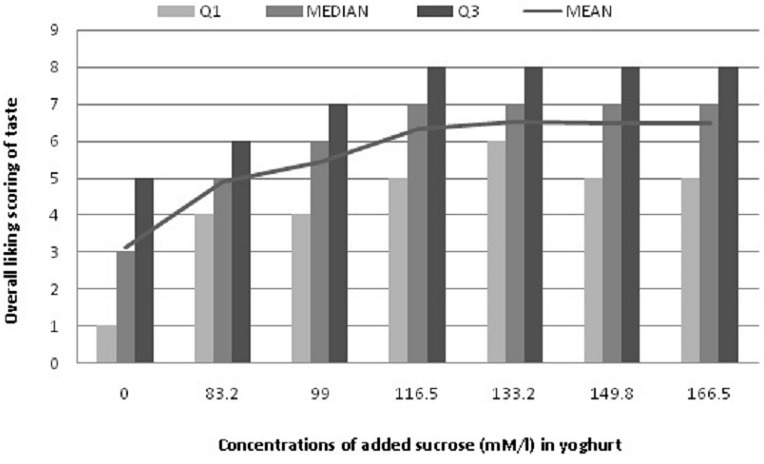
Participants distribution according to quartiles of their overall liking score of yogurt with varying sucrose concentration (n = 201) (Q1 = Quartile 1 and Q3 = Quartile 3)

**Overall liking**: Table 1 summarized taste scale results. Participants scored highest liking taste (81%) for yoghurt with 133.2mM/l, which corresponds to -20% reduction of added sugar (p < 0.05). The taste of yoghurt with 116.5mM/l (corresponding to -30% of the added sugar)) was liked by 74% of the participants. These findings were confirmed by the first quartile evolution (Q1) of hedonic test score by participants as presented in Figure 1. The highest Q1 taste score obtained were for the concentrations of 133.2 mM/l (Q1 = 7). Q1 for 116.5 mM/l was equal as for 149.8 and 166.5 mM/l, indicating that liking yogurt with sugar reduction at 30% was equal to 0%.

**Table 1:** Participants distribution according to their overall liking score of yogurt with varying sucrose concentration (n = 201)

	**Concentrations of added sucrose (mM/l)**							
**Liking scores**	0	83.2	99	116.5	133.2	149.8	166.5	*p-*value
I dislike	69.6	41.3	26.4	13.4	11.4	16.4	18.9	< 0.000
I neither like nor dislike	14.4	14.9	20.9	12.4	7.9	9.4	8.4	
I like	15.9	43.8	52.7	74.1	80.6	74.1	72.6	

Results are presented in percentage of total population by each concentration of added sugar p-value was calculated using Freidman’s Chi^2^ test = 366.114, test significance was set at a *p-*value < 0.05

**Figure 2:**
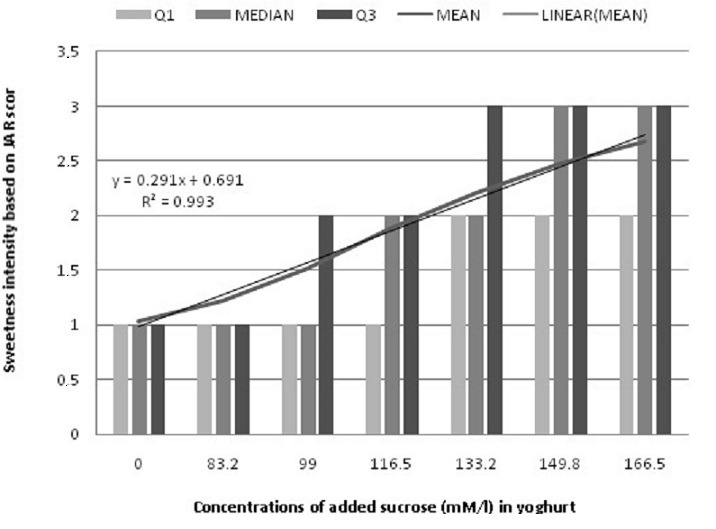
Participants distribution according to quartiles of JAR score of yogurt with varying sucrose concentration (n = 201) (Q1 = Quartile 1 and Q3 = Quartile 3)

**Sweetness liking**: 72.6% of participant indicated that yoghurt with a concentration of 166.5 mM sucrose/l (0% reduction) was too sweet versus 96.5% who reported that 0mM/l (100% reduction) was not sweet enough. 20% (133.2mM/l) and 30% reduction were considered as “Just about right” by 42.7% and 44.3% respectively (Table 2). Median first and third quartile of 0 and 83.2 mM sucrose/l were indicated by 50% of taste evaluators who scored them as not sweet enough (Figure 2). 75% and 25% evaluated the yoghurt at 133.2 mM/l as sweet and too sweet, respectively. A strong positive correlation (r = 0.93) was obtained between two variables (sweetness taste and JAR score).

**Table 2:** Participants distribution according to their JAR score of yogurt with varying sucrose concentration (n = 201)

	**Concentrations of added sucrose (mM/l)**							
**JAR scores**	0	83.2	99	116.5	133.2	149.8	166.5	p-value
A little sweet	96.5	79.6	60.2	33.8	17.9	11.4	4.9	< 0.000
Just about right	3.5	18.9	28.4	44.3	42.8	28.8	22.4	
Too sweet	0	1.5	11.4	21.9	39.3	59.7	72.6	

Results are presented in percentage of total population by each concentration of added sugar. p-value was calculated using Freidman’s Chi^2^ test = 779.64, test significance was set at a *p-*value < 0.05

**Purchase intent**: **Table 3** showed that the best average score of purchase intent was obtained for sucrose concentration of 149.8 mM/l. This finding was significant compared to the overall average (3.80 versus 3.25, respectively) (p-value = 0.043). More than one third of participants (36.8%) confirmed their intention to purchase the yoghurt with sucrose concentration of 166.5mmol/l (0% reduction), besides, 35.8% and 40.3% for yoghurt with sucrose concentration of 133.2 mM/l and 116.5 mM/l respectively. In contrast, yoghurt with high reduction in sucrose obtained important percentage of participants who reported no intention at all to purchase it (30% for 100% sucrose reduction).

**Table 3:** Participants distribution according to their purchase intent scoring of yogurt with varying sucrose concentration (n = 201)

	**Concentrations of added sucrose (mM/l)**							p-value
Purchase intent scale	0	83.2	99	116.5	133.2	149.8	166.5	
1.I would definitely not buy	59.2	30.3	21.4	12.4	12.4	11.4	19.9	P < 0.000
2. I would probably not buy	4.9	7.4	10.9	7.5	5.9	6.9	5.5	
3. I don’t know	5.5	15.4	21.4	9.9	9.9	6.5	9.4	
4. I would probably buy	10.9	20.9	24.4	40.3	35.8	40.3	28.4	
5. I would definitely buy	9.4	15.9	21.9	9.9	35.8	34.8	36.8

Results are presented in percentage of total population by each concentration of added sugar. p-value was calculated using Freidman’s Chi^2^ test = 263.07, test significance was set at a *p-*value < 0.05

## Discussion

Morocco is a country undergoing nutritional transition. Obesity and overweight are highly prevalent, affecting about 50% of adults and15% of children [19]. High sugar consumption by Moroccan consumers is considered as one of the most contributing factor to obesity and overweight. Indeed, national consumption exceeds 1 million of tons yearly, corresponding to 108 g/person/ day [20]. Thus, sugar reduction in processed food is a priority. The objective of this study was to evaluate acceptance of yoghurts available in the local market with different concentration levels of added sugar by Morocan populations using reference level of sucrose. Findings revealed that yoghurts with reduced sucrose concentrations at -20% and -30% were highly accepted by respondents. According to previous studies, high concentrations of sugar were scored with highest preference rate [4, 6, 21]. For example, in Finland, several hedonic tests demonstrated that highest percentage of taste evaluations were were obtained for yogurts containing 10% of added sugar. In contrast lowest percentages were obtained for lower sugar concentrations [22]. Currently, there is a growing trend of consumer preference toward yoghurt with reduced sugar content as reported by [12]. These authors studied acceptance level of sugar reduction in two flavored yoghurts (strawberry and coffee) in Switzerland. Their findings revealed that yoghurt containing 10% added sugar was scored as too sweet while 7% added sugar more acceptable to consumers. Similar findings were obtained among American consumers by Bayarri et al (2011) [11].

However, 5% of added sugar yoghurt was judged as not sweet enough. Most of respondents in this study stated that sweetness taste intensity of sweetened yoghurt available in local markets in Morocco is “too sweet”. In Switzerland, consumers too reported that yoghurt with 10% added sugar sold in Swiss markets is considered as too sweet [12]. A Finnish study also revealed similar results [5]. In contrast, 52% of consumers increasingly liked yoghurt as the sugar content was increased [11]. Similrary, Pohjanheimo and Sandell (2009) obtained highest scores for yoghurts with high sucrose concentrations among Finnish participants [23]. However, the same study showed that some consumers' preference was to have yoghurt without additives and less sweet. On the other hand, a research in the USA on liking level of natural or flavored liquid yoghurt and sweetness intensity taste identified three groups of consumers. The first group did not like yogurt with a high sugar concentration, the second group liked the yoghurt with medium to high concentrations, whereas with the third group sensory properties did not seem to influence on liking level [6]. Nowadays, several and continuous awareness campaigns on healthy eating are taking place in different cities of Morocco. The Moroccan population is gaining knowledge regarding the harmful effects of increased sugar intake on health. The participants who tested the yogurt in this study may have triggered in them an analytical process as described by Johansen et al, 2010 [21]. Indeed, it has been shown that Norwegian consumers who benefited more from nutrition education campaign liked less sweetened yoghurt [24]. This may explain the fact that a large percentage of participants declared that commercialized yoghurts are “too sweet”. This will lead for better acceptance of yogurts with less sugar content among Moroccan consumers in comparison to existing yoghurt in the market. Indeed, 35-40% of participants said they would probably or definitively buy yogurts containing -20% or even -30% of reduced sucrose.

## Conclusion

Finding of this study indicate that yoghurts containing -20% and -30% added sugar were highly accepted and highly scored as “Just about Right”, in addition to high intention to purchase as expressed by respondents. Using JAR scale revealed that the currently available yogurt in the local market is perceived as “too sweet”. Thus, advocating the dairy industry is needed to make them commit toward sugar reduction, in order to help achieving the national sugar reduction strategy in Morocco.

### What is known about this topic

- Strategy of reducing sugar in food in Morocco is conceived to reduce prevalence of non-communicable diseases;
- Yoghurt available in Moroccan market is considered as rich in sugar (12.1g/100g).

### What this study adds

- First study at national level aiming to reduce sugar consumption in food;
- Currently available yoghurt in Moroccan market is considered as very sweet by Moroccan consumers;
- Yoghurts with sugar reduction of 20% and 30% were highly liked by participants.

## Competing interests

The authors declare no competing interest.
